# Transgenic Soybeans Expressing Phosphatidylinositol-3-Phosphate-Binding Proteins Show Enhanced Resistance Against the Oomycete Pathogen *Phytophthora sojae*

**DOI:** 10.3389/fmicb.2022.923281

**Published:** 2022-06-16

**Authors:** Emily E. Helliwell, Peter Lafayette, Brent N. Kronmiller, Felipe Arredondo, Madeleine Duquette, Anna Co, Julio Vega-Arreguin, Stephanie S. Porter, Eli J. Borrego, Michael V. Kolomiets, Wayne A. Parrott, Brett M. Tyler

**Affiliations:** ^1^Department of Botany and Plant Pathology, Oregon State University, Corvallis, OR, United States; ^2^School of Biological Sciences, Washington State University, Vancouver, WA, United States; ^3^Department of Crop and Soil Sciences, University of Georgia, Athens, GA, United States; ^4^Escuela Nacional de Estudios Superiores – León, Universidad Nacional Autónoma de México, León, Mexico; ^5^Department of Plant Pathology and Microbiology, Texas A&M University, College Station, TX, United States; ^6^Thomas H. Gosnell School of Life Sciences, Rochester Institute of Technology, Rochester, NY, United States

**Keywords:** soybean, oomycetes, resistance gene, phosphatidylinositol-3-phosphate, *Phytophthora sojae*

## Abstract

Oomycete and fungal pathogens cause billions of dollars of damage to crops worldwide annually. Therefore, there remains a need for broad-spectrum resistance genes, especially ones that target pathogens but do not interfere with colonization by beneficial microbes. Motivated by evidence suggesting that phosphatidylinositol-3-phosphate (PI3P) may be involved in the delivery of some oomycete and fungal virulence effector proteins, we created stable transgenic soybean plants that express and secrete two different PI3P-binding proteins, GmPH1 and VAM7, in an effort to interfere with effector delivery and confer resistance. Soybean plants expressing the two PI3P-binding proteins exhibited reduced infection by the oomycete pathogen *Phytophthora sojae* compared to control lines. Measurements of nodulation by nitrogen-fixing mutualistic bacterium *Bradyrhizobium japonicum*, which does not produce PI3P, revealed that the two lines with the highest levels of *GmPH1* transcripts exhibited reductions in nodulation and in benefits from nodulation. Transcriptome and plant hormone measurements were made of soybean lines with the highest transcript levels of *GmPH1* and *VAM7*, as well as controls, following *P. sojae*- or mock-inoculation. The results revealed increased levels of infection-associated transcripts in the transgenic lines, compared to controls, even prior to *P. sojae* infection, suggesting that the plants were primed for increased defense. The lines with reduced nodulation exhibited elevated levels of jasmonate-isoleucine and of transcripts of a *JAR1* ortholog encoding jasmonate-isoleucine synthetase. However, lines expressing *VAM7* transgenes exhibited normal nodulation and no increases in jasmonate-isoleucine. Overall, together with previously published data from cacao and from *P. sojae* transformants, the data suggest that secretion of PI3P-binding proteins may confer disease resistance through a variety of mechanisms.

## Introduction

Like all eukaryotic organisms, plants have evolved to perceive and respond to a wide spectrum of microbes. These include pathogens, which invade and colonize to the overall detriment of host plants, as well as mutualists that provide benefits such as increased nutrition or protection against biotic or abiotic stress. Plants detect and respond to this wide range of microbes through complex networks of response pathways that integrate information about microbial signals, endogenous signals, and abiotic conditions (Wang et al., 2019). One class of microbial signals includes microbe-associated molecular patterns (also called pathogen-associated molecular patterns, PAMPs) that are widespread across diverse taxa. Well-characterized PAMPs include bacterial flagellin, and chitin from fungal cell walls. These PAMPs are perceived through binding to host plant pattern recognition receptors (PRRs) on the plasma membrane surface, which may trigger a cascade of responses including but not limited to production of defense-related hormones such as salicylic acid, jasmonic acid, and ethylene (Aerts et al., 2021), production of phytoalexins, and pathogenesis-related proteins (Wang et al., 2019). Collectively these responses have been termed PAMP-triggered immunity (PTI) or basal resistance (Jones and Dangl, 2006; Wang et al., 2019; Naveed et al., 2020). These responses are often sufficient to inhibit potential pathogens from colonizing plant tissue to a detrimental degree.

Successful pathogens can overcome PAMP-triggered defense responses to enter and colonize host cells through production of toxins or specialized effector proteins that can inhibit basal defense, as well as initiate metabolic, physiological, or morphological changes within host cells to facilitate colonization (Torto-Alalibo et al., 2010). Effector proteins may be targeted to the apoplast or to the host cell cytoplasm. These proteins are often specific to particular pathogen species or strains. An additional, highly effective layer of plant defense, called effector-triggered immunity (ETI), is initiated by the detection of pathogen effectors by cytoplasmic receptors (Wang et al., 2019). Detection of apoplastic effectors by cell surface receptors can also trigger an effective defense response (Wang et al., 2019). Many plant disease resistance genes identified by plant breeders encode these cell surface or cytoplasmic receptors. However, those resistance proteins typically recognize only specific effectors, rendering otherwise resistant plants vulnerable against pathogen strains that do not produce the recognized effector. For this reason, an important goal has been the search for conserved pathogen processes that could be inhibited to produce broad-spectrum immunity.

Oomycete pathogens produce a superfamily of cytoplasm-targeted effectors characterized by a highly conserved Arg-X-Leu-Arg (RXLR) motif located near the N-terminus (Jiang et al., 2008), which is required for movement into plant cells (Whisson et al., 2007; Dou et al., 2008; Kale et al., 2010; Song et al., 2013). Analysis of fungal effectors has suggested that some may utilize related motifs (Kale et al., 2010; Rafiqi et al., 2010; Plett et al., 2011). Furthermore, some research has suggested that RXLR and RXLR-like motifs may bind to the lipid phosphatidylinositol 3-phosphates (PI3P), as part of the process of delivering effectors inside the plant cell (Kale et al., 2010). That observation has motivated attempts to interfere with the interaction of effectors with PI3P by secretion of PI3P-binding proteins. Helliwell et al. (2016) showed that constitutive expression of a transgene composed of a PI3P-binding domain fused to a soybean PR1a secretory leader conferred increased resistance against both oomycetes (*Phytophthora tropicalis* and *Promecotheca palmivora*) and fungi (*Colletotrichum theobromicola*) in *Theobroma cacao* using both transient and stable transformation methods. Lu et al. (2013) showed that secretion of PI3P-binding proteins by *P. sojae* interfered with infection of soybean and that PI3P-binding proteins targeted the surface of *P. sojae* hyphae. Zhou et al. (2021) also showed that PI3P-binding proteins could target the surface of *Phytophthora* hyphae to confer improved disease resistance in transgenic soybean and potato plants.

In this study, we constructed stable transgenic soybean (*Glycine max*) plants expressing several lipid-binding proteins and control proteins. We observed that soybean plants expressing functional PI3P-binding proteins fused to a soybean PR1a secretory leader exhibited substantially increased resistance to *Phytophthora sojae* infection, though some, but not all, lines also exhibited reduced colonization by the mutualistic nitrogen-fixing bacterium *B. japonicum*, which is not known to produce PI3P. In the transgenic soybean plants expressing PI3P-binding proteins, measurements of the transcriptomes and of plant hormones suggested that the plants may be primed for elevated defense.

## Materials and Methods

### Generation and Validation of Soybean Transgenic Lines

The PI-P-binding domains fused to EGFP were amplified from pGH126gfp (Helliwell et al., 2016) with primers PR1a-F-Asc ATTAGGCGCGCCATGGGGTACATGTGCATTAAGA and eGF P-R-Avr, ATATCCTAGGTTACTTGTACAGCTCGTCCATGC and cloned into pGmUbiP between the soybean GmUbi3 promoter (Hernandez-Garcia et al., 2009) and the pea rbcS terminator (Coruzzi et al., 1984). The expression cassette was then moved into pSPH2 (Jacobs et al., 2015), which contains a hygromycin phosphotransferase gene under the control of the potato Ubi3 promoter and terminator for transgenic event selection. Transgenic soybeans were derived as described by Hancock et al. (2011). Events homozygous for the hph gene and null segregants were identified using Invader (Third Wave AgBio, Madison, WI) and verified with primers PR1a-F-Asc and eGFP-R-Avr.

### Inoculation With *Phytophthora sojae* and Quantification of Disease

For all inoculation conditions, six soybean seeds were sown in potting soil in 6-inch pots and maintained in growth chambers at 28°C light/24°C dark. For inoculation of detached soybean trifoliate leaves with zoospores, *P. sojae* strain P6497 (race 2) was inoculated on sterile 10% V8 agar and then incubated for 10 days at 23°C in the dark. Zoospores were produced by washing the resulting mycelia on the plates with sterile deionized water, every 30 min for 4 h, until zoospore release began. Plates were then flooded with 7 ml deionized water and incubated for 12–16 h at 14°C in the dark. The second trifoliate leaves were cut from 14-day-old soybean plants and placed in petri dishes containing a Kimwipe (Kimberly–Clark) wetted with 1 ml of sterile deionized water. Each leaflet of a trifoliate leaf was inoculated with a single drop of deionized water containing 200 zoospores (2 × 10^3^ zoospores per ml). Plates were sealed with parafilm and incubated for 4 days at room temperature, with 12-h dark and light. Disease severity was assessed by photographing each leaflet and measuring the area of each lesion using ImageJ (Schneider et al., 2012). To measure the relative amount of *P. sojae* biomass, inoculated leaflets were frozen in liquid nitrogen, and genomic DNA was extracted using a CTAB method (Helliwell et al., 2016). For quantitative real-time PCR (qRT-PCR), Takara SYBR green was used according to the manufacturer’s instructions, with 50 ng of genomic DNA per reaction with primers measuring housekeeping genes *G. max Cyclophilin 2* (*CYP2*) and *Phytophthora sojae Actin* (Wang et al., 2011). The ΔΔC_t_ method was used to calculate the relative quantity to *P. sojae* gDNA to *G. max* gDNA (Wang et al., 2011).

For hypocotyl inoculation, *P. sojae* P6497 was inoculated onto sterile 10% V8 agar and incubated in the conditions described above for 7 days. On the day of inoculation, soybean hypocotyls (on 10-day-old soybeans, six per pot) were wounded by gently scraping 0.5 cm between the soil line and the cotyledons, and a 2.5-mm^2^ square of *P. sojae* mycelia was placed, mycelial side down over the wounded area. Inoculated plants were maintained in a growth chamber at 28°C light/24°C dark for 3 days; then, plants were scored by measuring the lesion length and the percentage of collapsed plants per pot.

### Inoculation With *Bradyrhziobium japonicum* and Quantification of Nodulation

Soybean seeds were sterilized by exposure to chlorine gas for 6 h and then sown individually into 130-ml cone-shaped containers containing a sterile mix of 2:1 sand and SunGrow Sunshine #1 potting medium (Sun Gro Horticulture). *Bradyrhziobium japonicum* USDA 110 was maintained on plates containing modified arabinose gluconate (MAG) agar and inoculated into a 1-ml culture of MAG media and incubated at 28°C, 270 rpm for 48 h. The 1-ml cultures were added into a 24-ml volume of MAG media in a 50-ml Falcon tube and incubated at 29°C, 270 rpm for 72 h (modified from Sachs et al., 2011). *Bradyrhziobium japonicum* cells were harvested and resuspended in sterile 1/2X Jensen’s media at a concentration of 10^7^ cells/ml. Nine-day-old plants were inoculated by pipetting 1 ml of either the *B. japonicum* suspension or negative (1/2X Jensen’s buffer) at the base of the shoot. Plants were maintained in 28°C/24°C day/night greenhouse conditions for 4 weeks before harvest and data collection.

Just prior to harvest, a Soil Plant Analysis Development (SPAD) chlorophyll meter (Spectrum Technologies) was used to quantify leaf chlorophyll content by taking a reading from the center of the middle leaf in the 1st, 2nd, and 3rd trifoliate. The shoots were clipped off at the soil line, then placed into an envelope, and dried in a 60°C oven for 3 days before collecting shoot weight. Roots were washed to remove all planting substrate; then, the total number of nodules was counted per root. The size (diameter) was recorded for the largest three nodules on each root using a set of calipers.

### Assay of Soybean Transcript Levels by Quantitative Real-Time PCR

RNA was extracted from primary leaves of 10-day-old soybean seedlings using RNeasy (Qiagen) RNA extraction reagent according to the manufacturer’s instructions. cDNA synthesis was performed by pre-treating 1 μg RNA with DNAse I (Promega) at room temperature for 30 min. cDNA synthesis from RNA was performed using 50 ng oligo(dT) and New England Biolabs (NEB) M-MLV according to the manufacturer’s instructions. qRT-PCR was performed on an Applied Biosystems PRISM® 7500 FAST Sequence Detection System using Takara SYBR green according to the manufacturers’ instructions.

### RNA Sequencing and Transcriptomic Analysis

Six soybean seeds were sown per pot, seven pots per line per replicate. Pots were arranged in a randomized block design and maintained in a growth chamber under the conditions described above. Soybean hypocotyls were inoculated as described above, with four pots per line inoculated with *P. sojae* P6497 (*P. sojae*-inoculated), and three pots per line inoculated with a sterile 2.5-mm^2^ square of 10% clarified V8 agar (mock-inoculated). Hypocotyl segments, 1 cm long, centered on the inoculated areas were harvested 12 h later, pooling 18 plants each for mock- and *P. sojae*-inoculated lines, then snap-frozen in liquid nitrogen and stored in −80°C. One *P. sojae*-inoculated pot per line was maintained for an additional 3 days in the growth chamber to verify successful inoculation. The experiment was repeated independently three more times (for a total of four replicates), and the three most successful (determined by verification of disease development) replicates were used for RNA sequencing. RNA was extracted from each sample (pool of 18 hypocotyl segments per treatment per line) by the method described above, and 1 μg of total RNA sequenced on an Illumina HiSeq 3000, using a 150-base pair, paired-end module at the Oregon State University Center for Genome Research and Biocomputing. Supplementary Table 2 shows the detailed FASTQ report, including the numbers of passed filter reads, the yield, and the quality score for each demultiplexed RNA sample.

The *G. max* genome v1.1 (Schmutz et al., 2010) or the *P. sojae* genome v3.0 (Tyler et al., 2006) was indexed using TopHat (Trapnell et al., 2009), and the fastq files were aligned to the genome with Bowtie2 (Langmead et al., 2009). Count tables to quantify the number of reads per gene were generated with the SAMtools function htseq-count (Li et al., 2009). Normalization of the number of counts was run using EstimateSizeDisperson function, and PCA plots were conducted using the R package DESeq (Anders and Huber, 2010). A mixed model analysis, using replicate as a random effect, was run to determine differential expression with a significance cutoff of Benjamini–Hochberg-adjusted *p* < 0.1. The transcriptome data have been deposited in the NCBI Gene Expression Omnibus database accession GSE201739.

### Gene Ontology Annotations and Enrichment Analysis

Annotations of soybean genes of interest, including the identity of Arabidopsis orthologs, were obtained initially using AgriGO (Du et al., 2010). Then, each annotation was reviewed manually using information about the Arabidopsis ortholog obtained from The Arabidopsis Information resources (TAIR)^1^ version December 2021. Gene Ontology (GO) biological process annotations were transferred to the soybean genes from their Arabidopsis orthologs, and higher level parent terms such as “GO:0098542 Defense response to other organism” and “GO:0009725 Response to hormone” were added where appropriate in order to aggregate annotations. To calculate the enrichment of specific GO terms among particular soybean gene sets, the fraction of the soybean gene set carrying the annotation was compared to the fraction of the Arabidopsis genome containing the annotation, using Fisher’s exact test. The false discovery rate was controlled (adjusted *p* < 0.1) using the method of Benjamini and Hochberg (1995) based on the total number of GO terms represented in the soybean gene set of interest.

### Soybean Hormone Assays

Experimental design and soybean inoculation were carried out identically to that of the RNA sequencing experiment, except that only control and GmPH lines were included. In addition to harvesting and pooling soybean hypocotyls, primary leaves were collected and snap-frozen in liquid nitrogen and then stored at −80°C. Tissue was ground in liquid nitrogen, and 100 mg of frozen powder was placed in 2-ml screw-top microcentrifuge tubes. Phytohormone measurement and quantification were performed at Texas A&M University, with three independent biological replicates, two technical repeats per biological replicate. Phytohormone extractions followed Christensen et al. (2013), with the following modifications: 500 μl of phytohormone extraction buffer [1-propanol/water/HCl (2:1:0.002 v/v/v)] and 10 μl of 5 μM solution of deuterated internal standards: d-IAA [(2 H5) indole-3-acetic acid, Olchem], d-ABA [(2 H6)(+)-cis,trans-ABA; (Olchem)], and d-JA (2,4,4-d3, acetyl-2,2-d2 JA; CDN Isotopes), and d-SA (d6-SA, Sigma) was added to ~100 mg of ground mesocotyl tissue. Samples were agitated for 30 min at 4°C in dark conditions, and then, 500 μl of dichloromethane was added to the samples. The samples were agitated again for 30 min at 4°C and centrifuged at 13,000 × *g* for 5 min. To prevent autoxidation of reactive fatty acid derivatives, the lower layer collected into a glass vial was evaporated under a nitrogen gas stream to concentrate the extracted metabolites. The concentrated samples were resuspended in 150 μl of methanol and were centrifuged at 14,000 × *g* for 2 min to pellet any debris. One hundred microliters of supernatant was collected into an autosampler vial for direct injection into LC-ESI-MS/MS. The quantification of multiple hormones, oxylipins, and other defense-related metabolites utilized methods described in Wang et al. (2020). In particular, the quantification utilized an Ascentis Express C-18 Column (3 cm × 2.1 mm, 2.7 μm) connected to an API 3200 LC-electrospray ionization-tandem mass spectrometry (MS/MS) with multiple reaction monitoring (MRM). The injection volume was 2 μl and had a 600 μl/min mobile phase consisting of Solution A (0.05% acetic acid in water) and Solution B (0.05% acetic acid in acetonitrile) with a gradient consisting of (time–%B): 0.3%–1%, 2%–45%, 5%–100%, 8%–100%, 9%–1%, 11–stop. The metabolite and phytohormone m/z values and retention times are as listed in Wang et al. (2020). Statistical analysis was done using a one-way ANOVA with Duncan’s multiple range test, and letters represent significance groups at *p* < 0.05.

## Results

### Creation and Validation of Soybean Transgenic Lines

**Figure 1:**
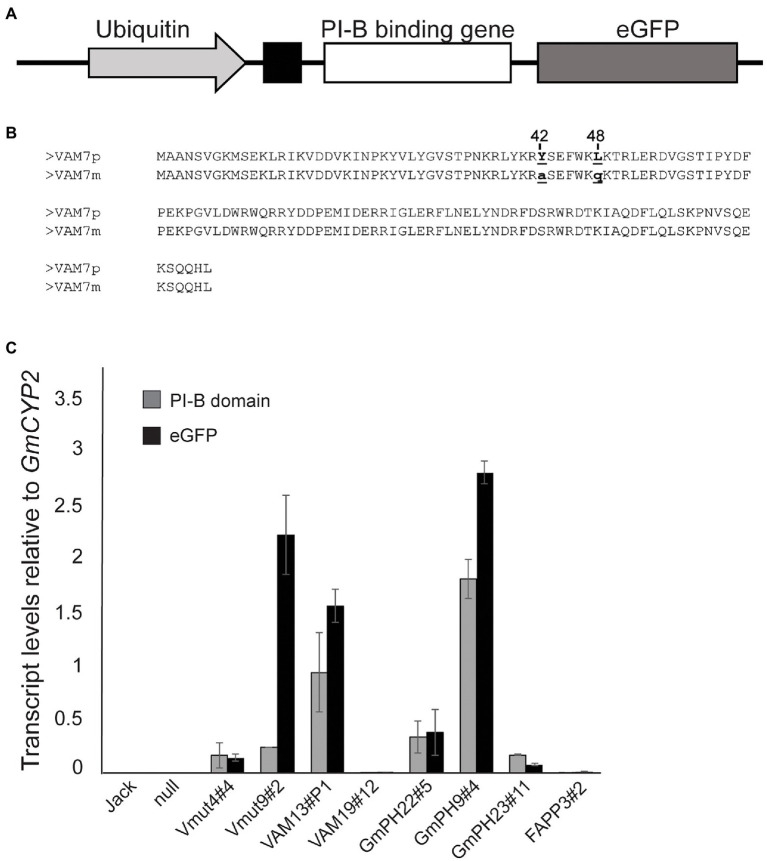
Structure and expression of transgenes encoding phosphoinositide-binding proteins. **(A)** Illustration of transgene showing the phosphoinositide-binding protein gene fused to the eGFP reporter gene, downstream of a PR1a secretory leader. The transgene is placed under control of the soybean *Ubiquitin 3* promoter. **(B)** Amino acid sequence alignment of the VAM7 PX domain compared to the non-PI3P-binding VAM7 mutant (Vmut). Amino acid substitutions from VAM7 to Vmut include tyrosine to alanine at position 42, and leucine to glutamine at position 48. **(C)** Transcript levels of transgenes across independent soybean lines, measured by quantitative reverse transcriptase PCR. Primer pairs specific for each PI-binding (PI-B) domain (e.g., VAM7, GmPH) and for the eGFP reporter domain were used in each case (Supplementary Table 6). Transcript levels are relative to the internal control gene *GmCYP2*. Bars are average of values from the trifoliates of three different plants, each trifoliate run in duplicate. Error bars show the SEM.

Transgene design and verification followed Helliwell et al. (2016). Three different phosphoinositide binding domains were chosen based on their differing specificities for PI3P and phosphatidylinositol-4-phosphate (PI4P). The pleckstrin homology (PH) domain from soybean GmPH1 (ortholog of AtPH1; Dowler et al., 2000), and the Phox homology (PX) domain from yeast VAM7 (Lee et al., 2006) were chosen for their specificity for PI3P. As a control, the PI4P-binding PH domain from human FAPP1 was chosen (Dowler et al., 2000; DiNitto and Lambright, 2006; Lemmon, 2008). All genes were fused to an enhanced GFP reporter gene at the 3’end and the secretory leader from the soybean PR1a protein (Cutt et al., 1988; Honée et al., 1998; van Esse et al., 2007) at the 5′ end to deliver the encoded protein to the apoplastic space. The gene fusions were placed under the control of the strong, constitutive soybean *Ubiquitin 3* promoter. An illustration of the transgene construct and comparative amino acid sequences of VAM7 and its non-binding mutant control (Vmut) are shown in Figures 1A,B. All recovered soybean transgenic lines exhibited normal growth, development and seed set.

Quantitative reverse transcriptase PCR (qRT-PCR) was used to determine transcript levels of each transgene in each of the homozygous T2 soybean lines. Using the *GmCYP2* housekeeping gene as an internal control (Jian et al., 2008), transcript levels of the specific PI-binding domain, along with the *GFP* reporter gene, were measured in each line. The results are shown in Figure 1C. As expected, there was no expression of any PI-binding protein gene, nor *GFP* reporter gene in the non-transgenic cv. Jack and null segregant line. Independent transgenic soybean lines expressing non-binding mutant *VAM* (Vmut4#4 and Vmut9#2) showed similar *VAM* transcript levels as each other (0.16 ± 0.01 and 0.25 ± 0.01, respectively), relative to *GmCYP2*. However, they exhibited vastly different *GFP* transcript levels (0.14 and 2.24), respectively, relative to *GmCYP2*, suggesting an aberrant transgene in Vmut9#2. Independent soybean lines expressing functional PI3P-binding *VAM* (VAM13#P1, VAM19#12) showed concordant *VAM* and *GFP* transcript levels, but the two differed greatly in the relative transcript levels of their *VAM*-*GFP* transgenes, with VAM13#P1 (*VAM* 0.94, *GFP* 1.57) much greater than VAM19#12 (*VAM* 0.0003, *GFP* 0.01). Similarly, the three independent soybean lines expressing functional PI3P-binding *GmPH* domain (GmPH22#5, GmPH9#4, and GmPH23#11) represented a range of *GmPH* and *GFP* expression. GmPH9#4 showed the highest relative transcript levels (*GmPH* 1.82, *GFP* 2.82) compared to GmPH22#8 (*GmPH* 0.33, *GFP* 0.38) and GmPH23#11 (*GmPH* 0.17, *GFP* 0.08). Lastly, the single line expressing functional PI4P-binding *FAPP* domain (FAPP3#2) showed low transcript levels of both *FAPP* (0.007) and *GFP* (0.016), relative to *GmCYP2*.

### Transgenic Soybeans Expressing Functional PI3P-Binding Proteins Showed Enhanced Resistance to a Virulent Isolate of *Phytophthora sojae*

**Figure 2:**
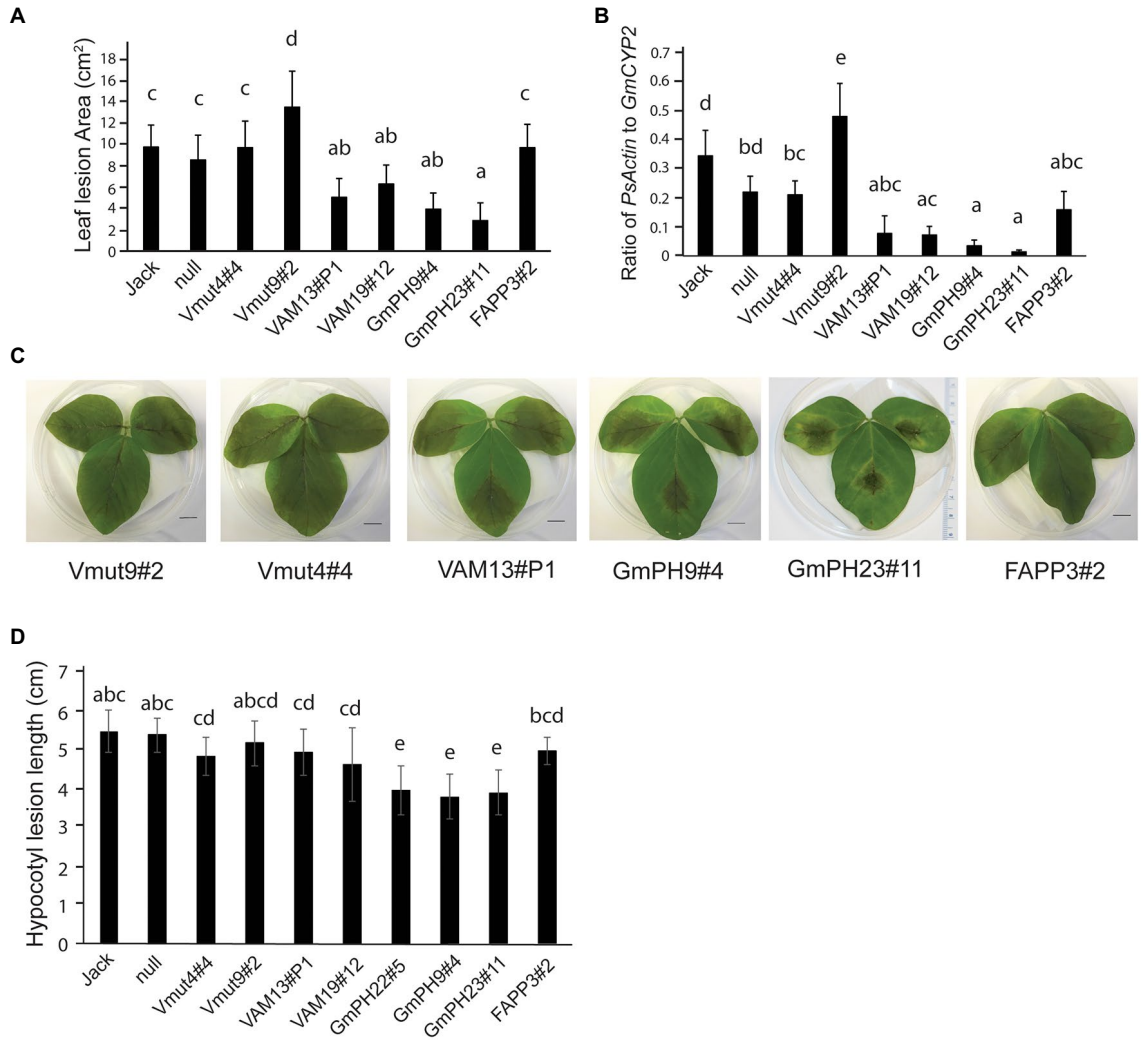
Inoculation of transgenic lines with *Phytophthora sojae*. **(A)** Lesion area measured 4 days after inoculation by zoospores of *P. sojae* strain P6497. Jack is the non-transformed cultivar and null is the null segregant originating from the VAM7-19 transgenic population **(B)** Ratio of *P. sojae* Actin genomic DNA to *Glycine max CYP2* genomic DNA in leaves, 4 days after inoculation by *P. sojae* zoospores. **(C)** Representative photographs of 2nd trifoliate leaves, 4 days after inoculation by *P. sojae* zoospores. **(D)** Length of *P. sojae* lesions 3 days after inoculation of soybean hypocotyls by mycelia. In **(A,B,D)**, letters represent different significance groups as measured by one-way ANOVA (*p* < 0.05). Letters represent different significance groups. Averages of 12 plants from each of three independent biological replicates are shown for all graphs; bars indicate SE.

To establish the susceptibility of transgenic soybeans expressing PI3P-binding proteins to a pathogenic oomycete, inoculation tests were conducted using both zoospores and mycelia of *P. sojae* strain, P6497. First, detached first trifoliate leaves were inoculated with *P. sojae* zoospores (Figures 2A,C). Independent lines expressing functional VAM7-GFP showed a significant partial reduction in lesion areas compared to the non-transformed control Jack (~35% and 50% for lines VAM19#12 and VAM13#P1, respectively; *p* < 0.05) 4 days after inoculation, with no significant difference between the two lines. Independent lines expressing GmPH-GFP showed a more substantial reduction in lesion area 4 days after inoculation (~65% and 71%, for lines GmPH9#4 and GmPH23#1, respectively; *p* < 0.05), with no significant difference between the two lines. Furthermore, statistical tests showed that there were no significant differences between non-transformed cv. Jack and a null segregant from the VAM7-19#12 transgenic population (“null”), one VAM7-non-binding mutant line (Vmut4#4), and the soybean line expressing a functional PI4P-binding FAPP protein. It is noteworthy to add that the second soybean line expressing a non-binding mutant (Vmut9#2) showed a hyper-susceptible response, with the resulting lesion area significantly larger (*p* < 0.05) than all other lines tested.

To confirm the lesion area measurements, qRT-PCR was used to determine the relative amount of *P. sojae* genomic DNA to soybean genomic DNA as a measure of pathogen proliferation (Figure 2B). Independent lines expressing functional VAM7-GFP showed a significant reduction in *P. sojae* biomass (77% for VAM13#P1, and 79% for VAM19#12; *p* < 0.05) as compared to non-transformed control cv. Jack. Independent lines expressing GmPH-GFP showed a very substantial reduction in *P. sojae* biomass (83% for line B, 96% for line C; *p* < 0.05) compared to non-transformed control cv. Jack. There were no significant differences among the FAPP3#2 line, null segregant, and non-binding VAM7 line Vmut4#4 (*p* > 0.05). Similarly to the lesion area measurements, non-binding VAM7 mutant line Vmut9#2 showed a significantly higher *P. sojae* biomass as compared to all of the other lines.

To test the effects of the transgenes on *P. sojae* stem infection, we performed hypocotyl inoculation tests (Figure 2D). As with the leaf assays, the soybean lines expressing GmPH PI3P-binding domains showed the smallest lesions with a significant 27%–30% reduction in lesion length. For the remainder of the transgenic lines, we saw more intermediate phenotypes. The soybean lines expressing functional VAM domains did not show significant differences from the soybeans expressing non-functional VAM mutants, or from soybeans expressing a functional PI4P-binding FAPP domain. However, this intermediate phenotype seen in the VAM, VAM mutant, and FAPP lines had a slight, yet significant reduction of 10%–15% in lesion length compared to the non-transgenic cv. Jack and null segregant lines.

Overall, the results of the infection assays indicated that the two transgenes encoding functional PI3P-binding proteins, *GmPH* and *VAM7*, could confer resistance to *P. sojae* compared to control lines, with the *GmPH-GFP* transgenes substantially more effective than the *VAM7-*GFP transgenes.

### Some Transgenic PI3P-Binding Soybean Lines Exhibit Reduced Colonization by Mutualistic *Bradyrhizobium japonicum*

**Figure 3:**
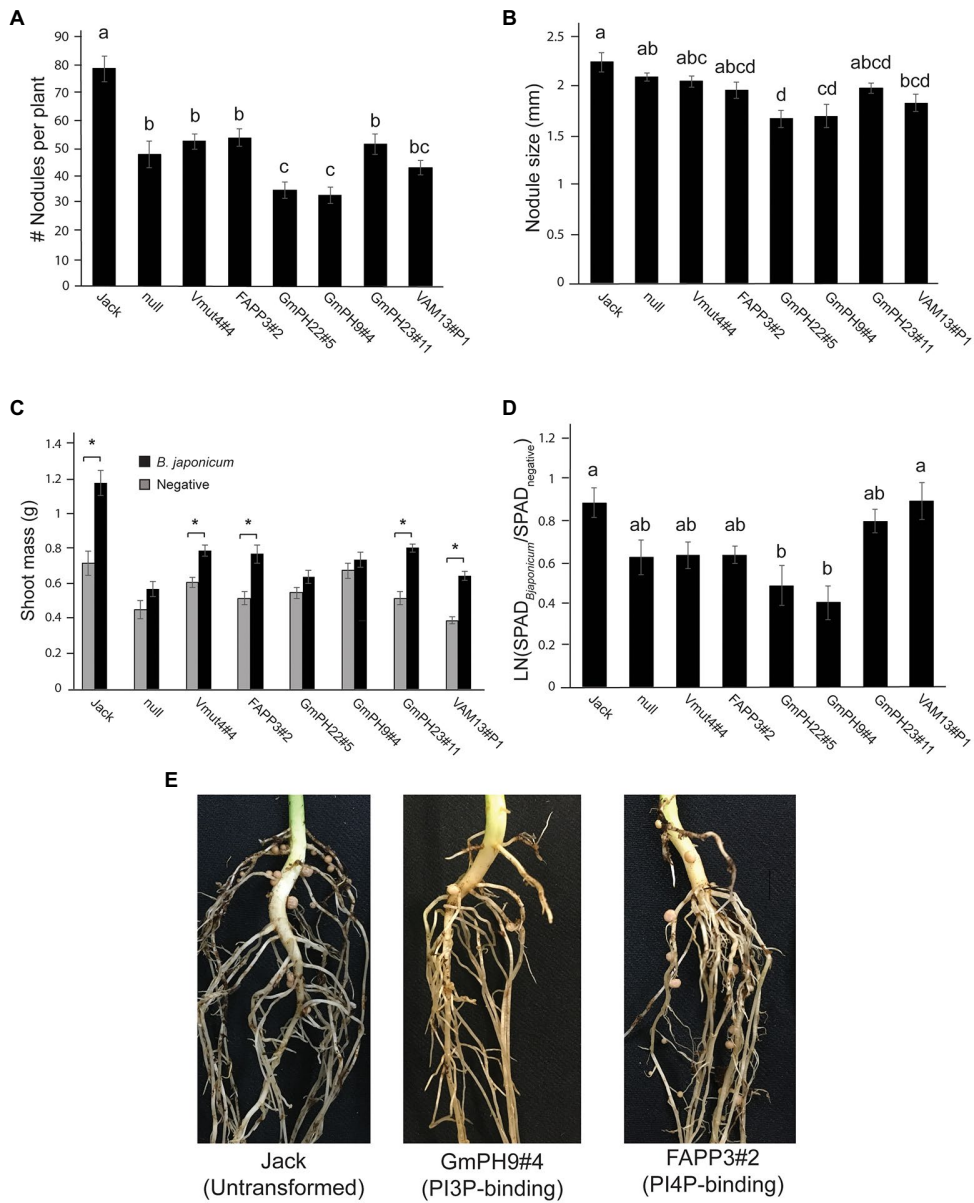
Effect of mutualistic nitrogen-fixing *Bradyrhizobium japonicum* USDA 110 on transgenic soybean lines. **(A)** Nodule count per plant. **(B)** Average size of the three largest nodules per plant. Data from **A** and **B** were analyzed using a one-way ANOVA with significance groups assigned by a Tukey HSD *post hoc* test, with significance threshold at *p* < 0.05. Each bar represents the average of 13 plants from a randomized block design. Error bars indicate the SD of the mean. **(C)** The average shoot mass for plants inoculated with sterile buffer (gray bars) or 10^6^ cells *B. japonicum* (black bars). Asterisks show the soybean lines where the shoot masses of the sterile-inoculated plants are significantly different than the *B. japonicum*-inoculated plants at a threshold of *p* < 0.05. **(D)** Response ratio of chlorophyll content of *B. japonicum*-inoculated plants relative to mock-inoculated plants. Measurements represent the log of the Soil Plant Analysis Development (SPAD) chlorophyll scores of inoculated plants over SPAD scores of mock-inoculated plants. Letters indicate significance groups as determined by a mixed model regression analysis followed by a Tukey HSD *post hoc* test. **(E)** Photographs show representative plants, 3 weeks post-inoculation.

Since in cacao, PI3P-binding domains conferred broad-spectrum resistance against oomycetes and fungi (Helliwell et al., 2016), and since colonization by mutualistic nitrogen-fixing bacteria is important for soybean growth and production, we conducted experiments in which the transgenic and control soybean lines were inoculated with mutualistic nitrogen-fixing *Bradyrhizobium japonicm*. The two GmPH-expressing lines with the highest transgene transcript levels (GmPH22#5 and GmPH9#4) exhibited significantly fewer nodules per plant than the control lines (non-transformed Jack, null segregant, transgenic non-binding mutant Vmut, PI4P-binding FAPP line; Figures 3A,E). These same PI3P-binding transgenic lines GmPH22#5 and GmPH9#4 showed a modest reduction in nodule size (21% compared to average of Jack and null; 15% relative to average of VAMmut and FAPP), but the only the difference with Jack and the null segregant line was statistically significant. GmPH23#11 showed no significant differences compared to the controls (Figures 3B,E). Data were also collected on the physiological responses of the soybean lines after inoculation by *B. japonicum* to gauge the benefits that the plant might be receiving from the mutualist. Dried shoot mass measurements were taken for sterile-inoculated and *B. japonium-*inoculated plants, and pairwise t-tests for each line showed that lines GmPh22#8 and GmPh9#4 failed to show a significant increase in shoot mass in *B. japonicum*-inoculated plants, in contrast to all other lines except the null segregant, and the strong transgenic GmPH22#8 and GmPH9#4 lines (Figure 3C). Chlorophyll content data collected with a SPAD meter from sterile-inoculated and *B. japonicum*-inoculated plants showed that lines GmPH22#5 and GmPH9#4 exhibited a significantly lower chlorophyll content response compared to the non-transformed cv. Jack (45 and 55% reduction, respectively; *p* < 0.05), but the differences with the other control lines (24 and 36% reduction, respectively) were not significant (Figure 3D). The remainder of the lines (non-binding Vmut, PI4P-binding FAPP, weak-expressing GmPH23#11) showed no significant differences (Figure 3D).

Taken together, these results revealed that expression of some PI3P-binding domains that confer *P. sojae* resistance also could increase resistance to colonization by a nitrogen-fixing symbiont, *B. japonicum*, despite the fact that *B. japonicum* does not produce PI3P.

### RNA Sequencing Analysis

Since the lines expressing PI3P-binding proteins appeared to exhibit resistance to *B. japonicum* as well as *P. sojae*, we conducted transcriptome measurements to assess the physiological states of the resistant lines compared to the control lines. In particular, we aimed to identify genes that might be elevated in both the GmPH and VAM lines compared to the controls, either in healthy or *P. sojae*-infected tissues. We also aimed to determine whether there were gene expression differences between the GmPH and VAM lines that might account for their differences in resistance levels and nodulation phenotypes. To identify an optimal early timepoint after inoculation of *P. sojae* to sample for RNA sequencing, we aimed to find a time point when there was abundant colonized tissue, but the host tissue was still largely intact.

Seedlings of susceptible cultivar Williams were inoculated with *P. sojae* P6497 mycelia on the hypocotyl and then sampled 0, 6, 12, 18, 24, and 48 h after inoculation. The earliest visible signs of infection appeared 18 h after inoculation, as evidenced by discoloration of the host tissue. Real-time quantitative PCR of genomic DNA was used to measure the ratio of *P. sojae* to soybean biomass in each sample (Supplemental Figure 1). The relative level of *P. sojae Actin* showed a 28-fold increase between 6 and 12 h after inoculation, followed by continued rapid proliferation of the pathogen. Therefore, we selected 12 h post-inoculation to sample the tissues for RNA sequencing.

**Figure 4:**
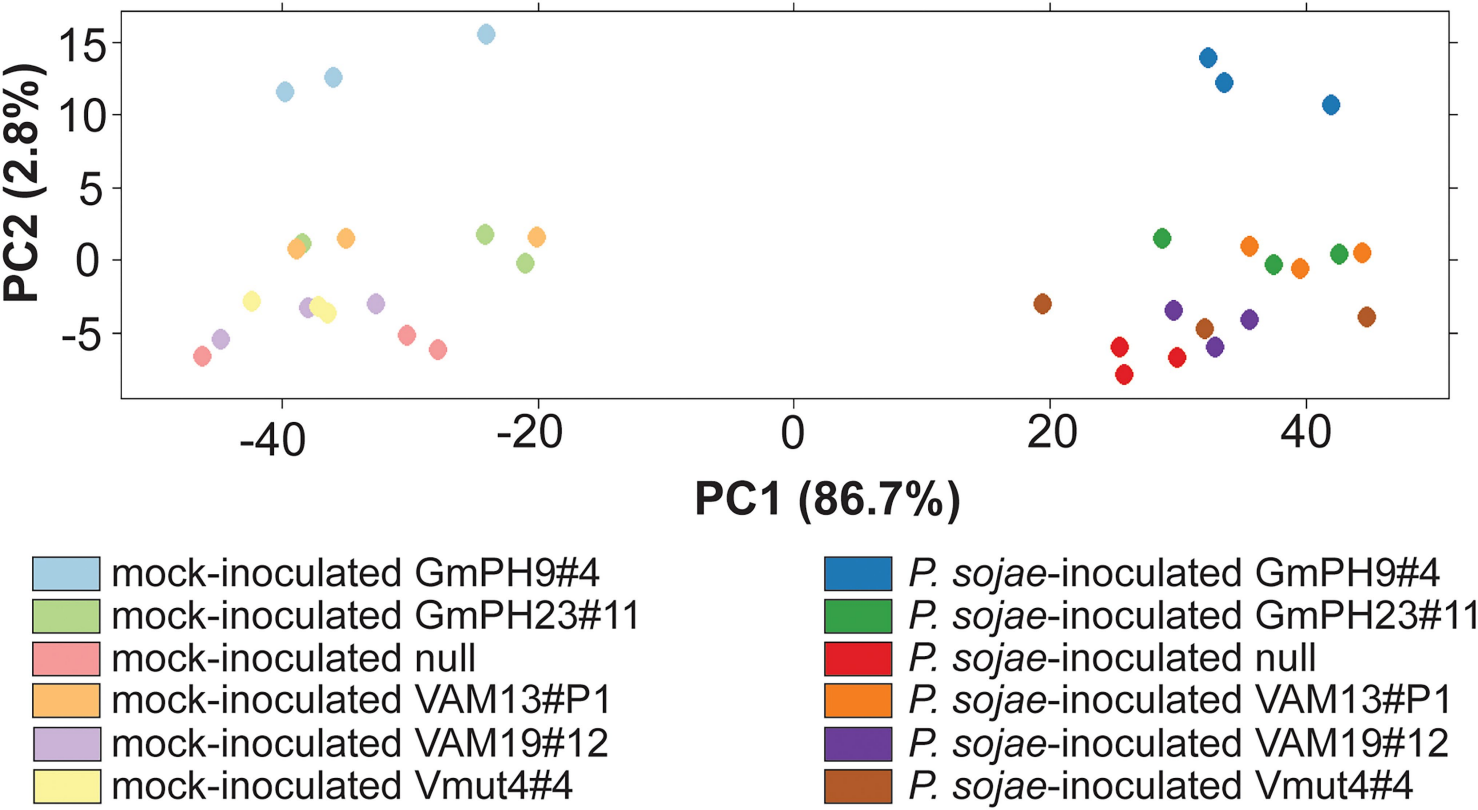
Principal component analysis of RNA sequencing count data generated by DEseq. Each dot represents one RNA library; there were three libraries per genotype-treatment replicate.

The design of the RNA sequencing experiment included two GmPH lines, two functional VAM lines, and the Vmut and null as controls (Supplementary Table 1). The sequences (150 bp paired ends) were aligned to *G. max* genome V1.1 (Phytozome id360 V9.0; Supplementary Figure 2). Principal components analysis (Figure 4) was used to identify the major sources of variation among the transcriptomes. As expected, *P. sojae* infection accounted for most variation (PC1, 86.7% of variation). PC2 (2.8% of variation) accounted for much of the differences associated with the type of transgene. In particular, the highest-expressing GmPH line (GmPH9#4) clearly separated from the other lines, both with and without *P.sojae* infection. The lower-expressing GmPH line (GmPH23#11) and the higher expression VAM line (VAM13#P1) also consistently separated from the controls under both treatments. The non-transformed null segregant, and the VAM mutant line, but also the lower-expressing VAM line (VAM19#12) were indistinguishable within each treatment. For subsequent analyses, data from the non-transformed null segregant line and transgenic non-binding VAM (Vmut) lines were combined as the “Control,” the two independent VAM lines (VAM13#P1 and VAM19#12) were combined into genotype “VAM,” and the two independent GmPH lines (GmPH9#4, GmPH23#11) were combined into genotype “GmPH.”

**Figure 5:**
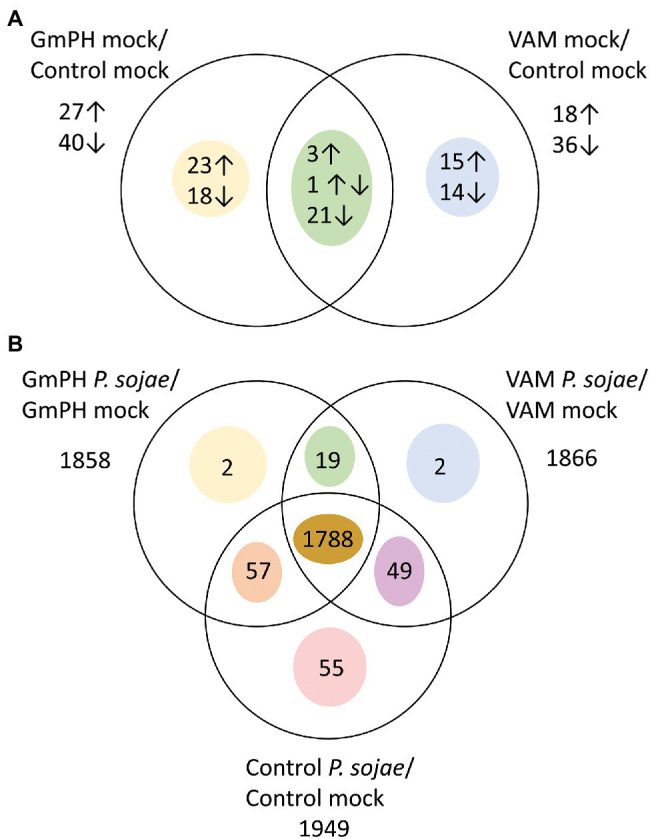
Genes differentially expressed among transgenic lines following mock or *P. sojae* inoculation. **(A)** Numbers of genes with transcript levels differing significantly (FDR *p* < 0.1) and by at least 2-fold comparing GmPH or VAM lines with control lines. Arrows indicate whether transcript levels were higher or lower than the control lines. One gene had higher levels in GmPH lines but lower in the VAM lines. **(B)** Numbers of genes with transcript levels differing significantly (FDR *p* < 0.1) and by at least 2-fold comparing *P. sojae*- with mock-inoculated tissue.

To calculate differential expression, we used a mixed model analysis to compare Genotype (VAM or GmPH compared to Control), Treatment (*P. sojae* compared to Mock), and Genotype X Treatment interaction as fixed effects, with Replicate as a random effect. Effects were estimated for 34,527 out of 73,320 genes (47.1%) in the *G. max* v1.1 transcriptome. Of the genes not analyzed, 93% exhibited 0 counts for all conditions. The false discovery rate threshold was set at *p* < 0.1 using the method of Benjamini and Hochberg (1995). We added an additional threshold of a 2-fold difference based on likely biological relevance. The numbers of genes exhibiting significant differences and at least 2-fold differences are summarized in Figure 5.

To validate the results of the differential expression analysis of the RNA sequencing data, quantitative reverse-transcriptase PCR was used to measure a small sample of genes that tested as significantly different in the mock-treated GmPH control group, relative to the internal control gene *GmCYP2* (Glyma12g02790), which consistently showed a negligible change across genotypes and treatments. Four genes with different expression patterns were tested, namely Glyma04g02230, Glyma10g26320, Glyma02g14630, and Glyma18g07396. The results revealed good agreement between the estimates of transcript level changes from the RNAseq and qRT-PCR measurements (Supplementary Figure 3).

### Transcript Levels Altered by GmPH and VAM Transgenes in the Absence of Infection

In mock-inoculated tissues, 67 genes were significantly changed by 2-fold or more in the GmPH lines compared to the controls, whereas 54 were changed in the VAM lines (Figure 5A; Supplementary Table 3). However, only 25 genes were changed in both GmPH and VAM lines. Of these 25, only three were significantly elevated more than 2-fold in both sets of lines, while 21 were decreased in both sets of lines (Table 1). One of the genes elevated in both lines (155-fold in the GmPH lines and 6.4-fold on the VAM lines) was *Glyma17g19845*, which encodes a dihydroflavonol 4-reductase involved in anthocyanin biosynthesis. One of the genes decreased in both sets of lines was Glyma06g21480 which encodes an ortholog of the *Arabidopsis* ENHANCED DISEASE RESISTANCE 2 (EDR2), which is a negative regulator of cell death elicited by pathogen attack. Of note, a single gene was significantly elevated in the GmPH lines but decreased in the VAM lines (5.3-fold and 3.2-fold, respectively, resulting in a 17-fold difference between the two sets of lines). This gene, Glyma06g37401, encoded an ortholog of the *Arabidopsis* protein JAR1, which is responsible for the synthesis of the key defense signal jasmonate-isoleucine.

**Table 1:** Transcript-level differences in the absence of *Phytophthora sojae*.

Transcript-level differences in the absence of *P. sojae*	Number of genes
Mean (GmPH,VAM)/control >2x	3
Mean (GmPH,VAM)/control <0.5x	21
Total	24
GmPH/VAM >2x	26
GmPH more increased than VAM	15
GmPH less decreased than VAM	11
VAM/GmPH >2x	23
VAM more increased than GmPH	8
VAM less decreased than GmPH	15
Total	49

There were 41 genes significantly changed more than 2-fold in the GmPH lines but not the VAM lines (Figure 5A), including three glycosyl hydrolases that were elevated 3.2- to 4.3-fold. Among 29 genes significantly changed more than 2-fold in the VAM lines but not the GmPH lines, there were three transcription factors including *Glyma02g00870* (elevated 2.1-fold) which encodes an ortholog of the Arabidopsis ETHYLENE-RESPONSE FACTOR 1 (AtERF1). Also included was *Glyma14g12210* (elevated 4.6-fold) which encodes an ortholog of the *Arabidopisis* CULLIN 1, subunit of an SCF ubiquitin ligase complex involved in mediating responses to auxin and jasmonic acid. A third gene was *Glyma19g38740* (elevated 18-fold), which encodes an ortholog of *Arabidopisis* PUB22, an E3 ubiquitin ligase that negatively regulates plant immunity.

### Transcript Levels Altered in Both GmPH and VAM Lines in the Presence of *Phytophthora sojae*

In *P. sojae*-inoculated tissues, a total of 1972 gene transcript levels were significantly altered (FDR-adjusted *p* < 0.1) compared to mock inoculation by 2-fold or more in at least one of the GmPH, VAM, or control tissues (Figure 5B; Supplementary Table 4). Of those genes, 1788 were altered in all three genotypes. Only 19 genes exhibited transcript levels changed in both GmPH and VAM lines but not in the controls, whereas transcript levels of 55 genes were changed in the controls but not in either of the GmPH and VAM lines. To better explore the similarities in the GmPH and VAM lines’ responses to *P. sojae* infection, we selected all genes significantly altered by infection in both the GmPH and VAM lines, or only in the controls, and calculated those in which the geometric mean response to infection in the GmPH and VAM lines was at least 2-fold different than in the control lines. There were 43 genes in which the mean of the two lines was at least 2-fold greater than in the control lines and 14 in which the mean was at least 2-fold less (Table 2).

**Table 2:** Common transcript-level changes in the presence of *Phytophthora sojae*.

Transcript-level changes in the presence of *P. sojae*	Number of genes
Mean (GmPH,VAM)/control >2x	43
GmPH, VAM more increased	22
GmPH, VAM less decreased	9
GmPH, VAM unchanged; control decreased	12
Mean (GmPH,VAM)/control <0.5x	14
GmPH, VAM less increased	4
GmPH, VAM more decreased	4
GmPH, VAM unchanged; control increased	6
Total	57

### Transcript Levels Differentially Altered in GmPH and VAM Lines in the Presence of *Phytophthora sojae*

In order to explore differences in the transcript profiles of GmPH lines compared to VAM lines that might be associated with the differences in resistance of the lines to *P. sojae* infection, we identified genes in which transcript levels during *P. sojae* infection were more than 2-fold different between the two sets of lines, as well as significantly elevated (FDR-adjusted value of *p* < 0.1) during *P. sojae* infection in at least one of the two sets of lines. There were 22 genes with transcripts more strongly elevated during *P. sojae* infection of GmPH lines than of VAM lines, and 30 genes for which the reverse was true (Table 3). The 22 genes included *Glyma13g17340* (elevated 161-fold by infection in the GmPH lines, but only 73-fold in the VAM lines), which is an ortholog of the Arabidopsis *FMO1* gene involved in biosynthesis of L-pipecolic acid, a long-distance signal of systemic acquired resistance. The 30 genes more strongly elevated in the VAM lines included eight transcription factors, four in the myb class, three basic helix–loop–helix factors, and one C_2_H_2_ domain factor.

**Table 3:** Differential transcript-level changes in the presence of *Phytophthora sojae.*

Transcript-level changes in the presence of *P. sojae*	Number of genes
GmPH/VAM >2x	22
GmPH more increased than VAM	16
GmPH less decreased than VAM	6
VAM/GmPH >2x	30
VAM more increased than GmPH	25
VAM less decreased than GmPH	5
Total	52

### Gene Ontology Annotations of Genes Affected by Transgene Expression in the Absence of *Phytophthora sojae*

In order to explore the biological significance of the transcript-level changes noted above, we performed Gene Ontology (GO) annotation enrichment analysis. We compared the fraction of significantly changed transcripts annotated with each GO term with the overall frequency of genes annotated with that GO term within the Arabidopsis database used to annotate the soybean genes. We used Fisher’s exact test with false discovery rate control of 0.1 to identify annotation terms significantly enriched among each soybean gene set of interest.

Among 96 transcript levels significantly altered (FDR *p* < 0.1) by at least 2-fold in the GmPH or VAM lines compared to the control lines, in the absence of *P. sojae*, there were 10 in which the *Arabidopsis* ortholog was annotated with the GO term “GO:0098542 Defense response to other organism” (3.5-fold enrichment; FDR *p* < 0.1), 11 annotated with “GO:0009725 Response to hormone” (3.0-fold enrichment; FDR *p* < 0.1), and four genes annotated with “GO:0009753 Response to Jasmonic Acid” (7.3-fold enrichment; FDR *p* < 0.1). However, neither of these terms were significantly enriched among the 24 genes altered in both the GmPH and VAM lines (Table 4).

**Table 4:** Enrichment of Gene Ontology (GO) terms among genes significantly altered in transcript levels in GmPH and VAM lines in the absence of *Phytophthora sojae*.

Gene transcript changes	Altered in VAM or GmPH lines^b^		Altered in both VAM and GmPH lines^c^		Differing between VAM and GmPH lines^d^	
Significant genes^a^	(96 Genes)		(24 Genes)		(49 Genes)	
GO term or other treatment	Genes^e^	Enrichment^f^	Genes	Enrichment	Genes	Enrichment
GO:0098542 Defense response to other organism	10	3.5^*^	1	1.4	9	6.1^**^
GO:0050832 Defense response to fungus	4	2.5	1	2.5	2	2.5
GO:0042742 Defense response to bacterium	2	1.8	0	0	2	3.4
GO:0009725 Response to hormone	11	3.0^*^	2	2.2	7	3.8^*^
GO:0009753 Response to jasmonic acid	4	7.3^*^	0	0	3	10.7^*^
GO:0009737 Response to abscisic acid	2	1.4	0	0	2	2.7
GO:0009723 Response to ethylene	3	6.9	1	9.1	0	0.0
GO:0009735 Response to cytokinin	2	3.2	1	0	1	3.0
GO:0009733 Response to auxin	1	1.0	0	0	1	1.9
GO:0009751 Response to salicylic acid	1	1.8	1	7.0	0	0.0
GO:0009416 Response to light stimulus	4	5.7	3	17.1^*^	0	0.0
GO:0006979 Response to oxidative stress	3	3.1	0	0	3	6.0
GO:0009611 Response to wounding	2	3.0	0	0	1	0.0
Changed during *P. sojae* infection of control lines^g^	21	3.9^***^	4	3.0^*^	8	2.9^**^

a Detailed list of genes, annotations, and transcript levels are provided in Supplementary Table 2.

b Significantly (FDR-adjusted value of *p* < 0.1) elevated or decreased at least 2-fold in the mock-inoculated GmPH lines or VAM lines compared to the control lines.

c Genes that showed significant (FDR-adjusted value of *p* < 0.1) transcript changes of at least 2-fold in the same direction in both the GmPH and VAM lines compared to the control lines.

d Genes in which transcript levels differed between the GmPH and VAM lines by at least 2-fold and in which at least one set of lines (GmPH or VAM) differed significantly from the control lines.

e Number of genes meeting the criterion that were annotated with each GO term.

f The fraction of all significantly altered genes with the GO annotation, divided by the fraction of all genes with the GO annotation; asterisks indicate whether the enrichment is statistically significant based on a Fisher’s exact test with a false discovery rate correction, * FDR-adjusted *p* < 0.1; ** FDR-adjusted *p* < 0.001; *** FDR-adjusted *p* < 0.001.

g Transcript levels changed significantly (FDR-adjusted value of *p* < 0.1) by at least 2-fold in *P. sojae*-inoculated control lines compared to mock-inoculated control lines.

Among 49 transcripts differing in level by more than 2-fold between the GmPH and VAM lines (Table 1), there were nine in which the *Arabidopsis* ortholog was annotated with the GO term “GO:0098542 Defense response to other organism” (6.1-fold enrichment; FDR *p* < 0.01), 7 annotated with “GO:0009725 Response to hormone” (3.8-fold enrichment; FDR *p* < 0.1), and 3 genes annotated with “GO:0009753 Response to Jasmonic Acid” (10.7-fold enrichment; FDR *p* < 0.1; Table 4).

**Figure 6:**
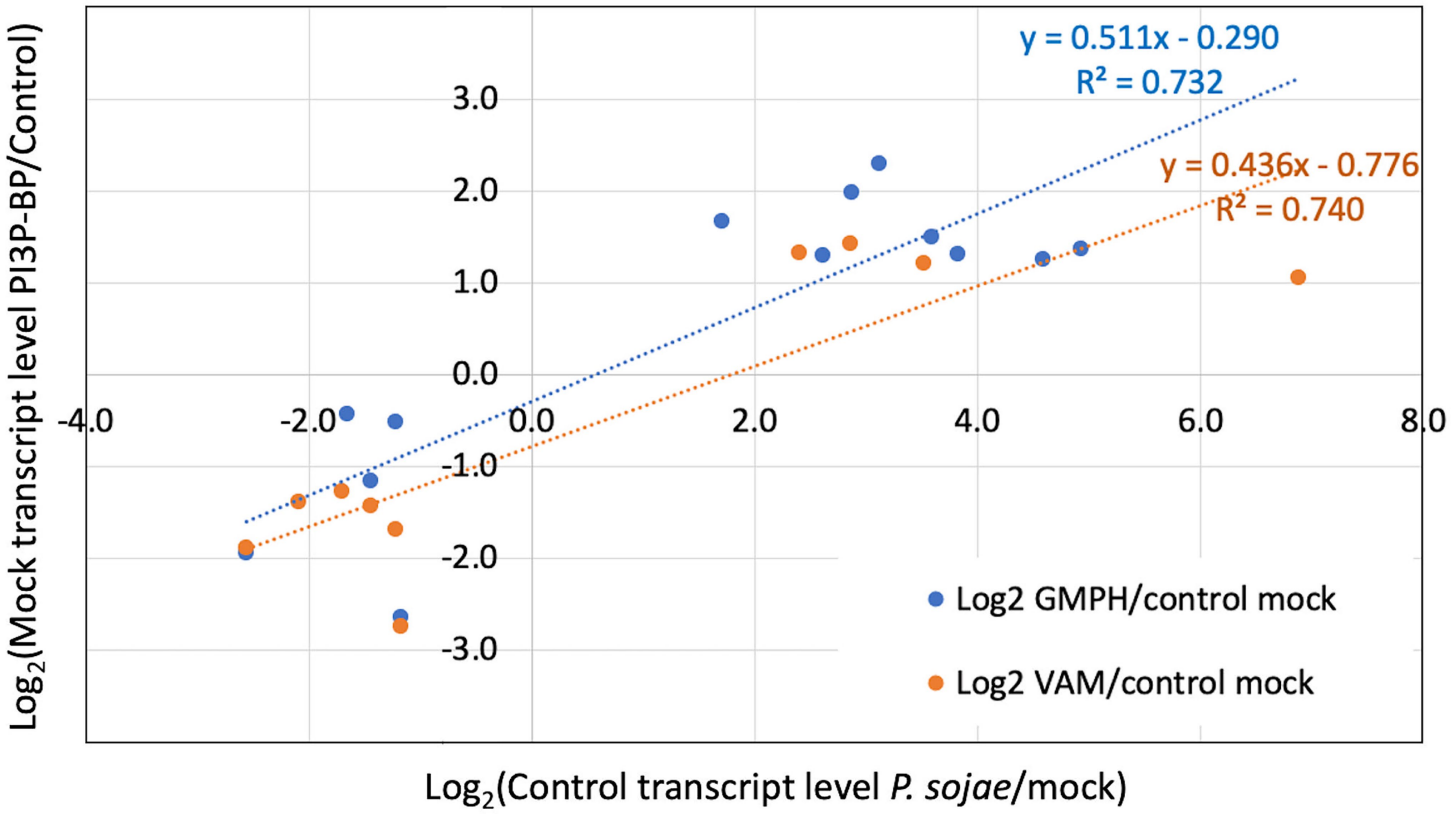
Correlation of relative transcript levels in mock inoculated GmPH and VAM lines with relative transcript levels in *P. sojae*-inoculated control lines. Only genes exhibiting significant changes in both Mock GmPH/Control and *P. sojae*/Mock Control or both Mock VAM/Control and *P. sojae*/Mock Control are included.

We also performed enrichment analysis on each gene set to determine the overlap with genes significantly altered by *P. sojae* infection of the control lines, to determine whether the expression of the GmPH and/or VAM transgenes was causing infection-associated genes to be altered. All three gene sets noted above exhibited significant enrichment for genes also altered by *P. sojae* infection of the control lines (2.9- to 3.9-fold, FDR *p* < 0.1; Table 4). Furthermore, as shown in Figure 6, there was a strong correlation (*R*^2^ > 0.7) between the changes induced by transgene expression in the absence of *P. sojae* with the changes induced by *P. sojae* infection of the control lines.

### Gene Ontology Annotations of Genes Affected by Transgene Expression in the Presence of *Phytophthora sojae*

As expected, infection-related GO terms were significantly enriched among the 57 genes in which the mean response of the GmPH and VAM lines to *P. sojae* infection was >2-fold different than the control lines. Those terms included “GO:0098542 Defense response to other organism,” “GO:0050832 Defense response to fungus,” and “GO:0006979 Response to oxidative stress” (Table 5). Also significantly enriched were genes annotated with “GO:0009725 Response to hormone” (8.4-fold, FDR *p* < 0.001) including genes annotated with “GO:0009753 Response to jasmonic acid” (18.4-fold, FDR *p* < 0.001) and “GO:0009737 Response to abscisic acid” (7.1-fold, FDR *p* < 0.01; Table 5).

**Table 5:** Enrichment of GO terms among genes significantly altered in transcript levels in GmPH and VAM lines in the presence of *Phytophthora sojae*.

Gene transcript changes	Mean response of VAM and GmPH lines different than controls^a^		Differing response between VAM and GmPH lines^b^	
Significant genes	(57 Genes)		(52 Genes)	
GO term	Genes^c^	Enrichment^d^	Genes	Enrichment
GO:0098542 Defense response to other organism	12	7.0^***^	11	7.1^***^
GO:0050832 Defense response to fungus	7	7.5^***^	6	7.0^**^
GO:0042742 Defense response to bacterium	6	8.9^**^	5	8.1^**^
GO:0009725 Response to hormone	18	8.4^***^	8	4.1^*^
GO:0009753 Response to jasmonic acid	6	18.4^***^	1	3.4
GO:0009739 Response to gibberellin	0	0.0	2	9.7
GO:0009737 Response to abscisic acid	6	7.1^**^	1	1.3
GO:0009723 Response to ethylene	2	7.7	0	0.0
GO:0009735 Response to cytokinin	1	2.6	0	0.0
GO:0009733 Response to auxin	2	3.3	1	1.8
GO:0009751 Response to salicylic acid	2	5.9	1	3.2
GO:0009416 Response to light stimulus	4	9.6^**^	3	7.9^*^
GO:0006979 Response to oxidative stress	4	6.9^*^	3	5.7
GO:0009611 Response to wounding	3	7.4^*^	0	0.0

Gene transcript criteria were as follows:

a Genes which showed significant (FDR-adjusted value of *p* < 0.1) transcript changes of at least 2-fold in the same direction in both the GmPH and VAM lines in response to *P. sojae* and in which the mean change of the GmPH and VAM lines was at least 2-fold different compared to the control lines, also includes genes in which the response in the control lines was at least 2-fold and significant (FDR-adjusted value of *p* < 0.1), but the GmPH and VAM lines both did not respond significantly.

b Genes in which changes in transcript levels in response to *P. sojae* infection differed between the GmPH and VAM lines by at least 2-fold and in which at least one set of lines (GmPH or VAM) differed significantly (FDR-adjusted value of *p* < 0.1) by at least 2-fold from the control lines.

c Number of genes meeting the criterion that were annotated with each GO term.

d The fraction of all significantly altered genes with the GO annotation, divided by the fraction of all genes with the GO annotation; asterisks indicated whether the enrichment is statistically significant based on a Fisher’s exact test with a false discovery rate correction: * FDR-adjusted *p* < 0.1; ** FDR-adjusted *p* < 0.01; *** FDR-adjusted *p* < 0.001.

Similarly, among the 52 genes in which the response to infection differed between the GmPH and VAM lines, “GO:0098542 Defense response to other organism” (6.4-fold, FDR *p* < 0.001) and “GO:0009725 Response to hormone” (4.6-fold, *p* < 0.01) were significantly enriched (Table 5). However, “GO:0009753 Response to jasmonic acid” (3.4-fold, FDR *p* > 0.1) was not significantly enriched (Table 5).

### Transgenic Soybeans Expressing GmPH Domains Show Changes in Levels of Phytohormones Involved in Defense Signaling

Given the significant enrichment of genes annotated with “GO:0009725 Response to hormone” among the transcriptome data, we assessed differences in phytohormone production in the mock-and *P. sojae*-inoculated GmPH and control soybean lines, using a similar experimental design as for the RNA sequencing experiment. After inoculation of hypocotyls, both infected hypocotyl tissue and uninfected primary leaves were harvested 12 h later and hormones measured with LC-electrospray ionization-tandem mass spectrometry (LC-ESI-MS/MS). The full set of phytohormone measurements is listed in Supplementary Table 5.

**Figure 7:**
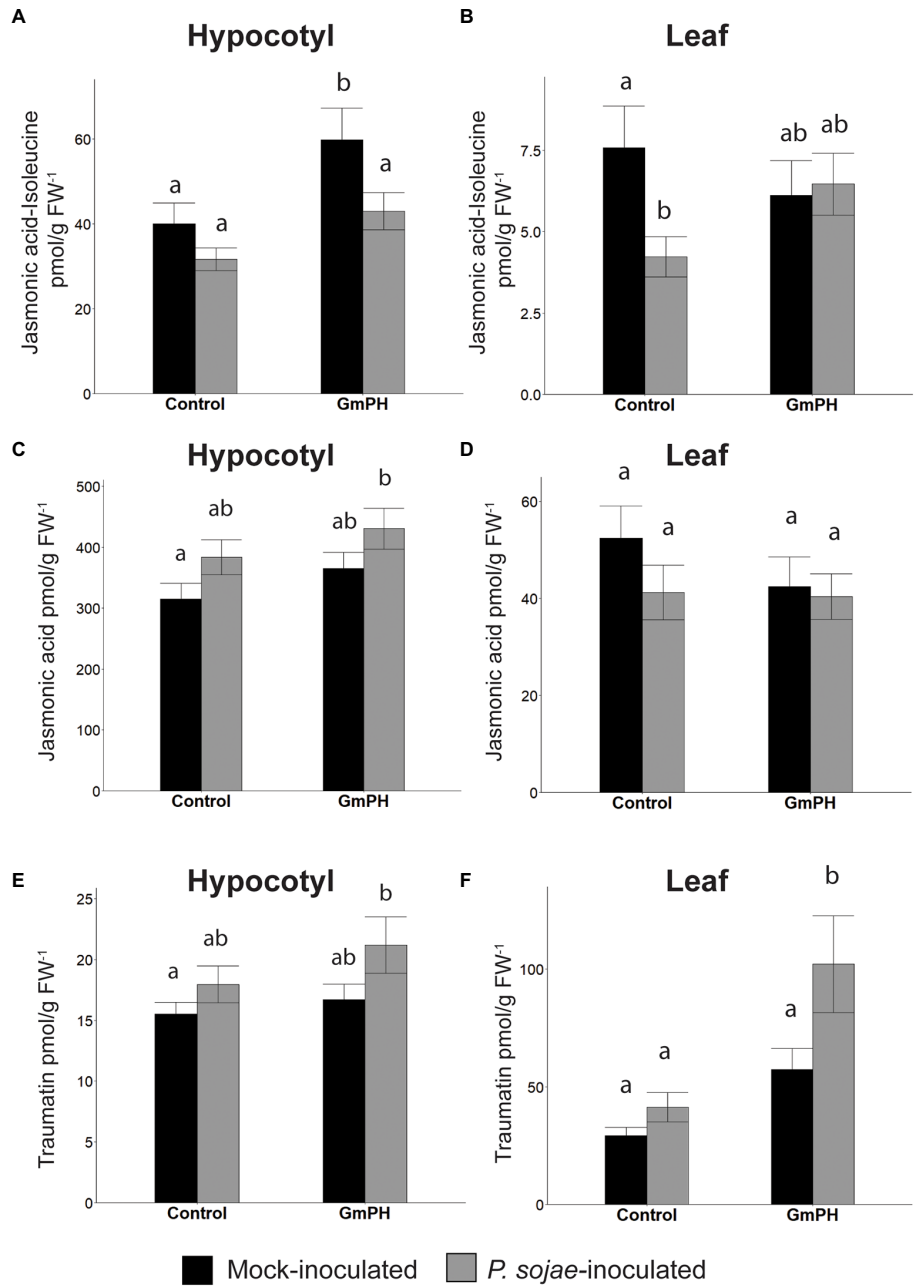
Phytohormone levels in GmPH and Control soybean lines. **(A,B)** Levels of Jasmonic acid-Isoleucine conjugate in hypocotyls **(A)** or primary leaves **(B)** of control (null and Vmut) and GmPH (GmPH9#4 and GmPH23#11) lines after mock- or *P. sojae* inoculation. **(C,D)** Levels of jasmonic acid, in hypocotyls **(C)** or primary leaves **(D)** of control and GmPH lines after mock- or *P. sojae* inoculation. **(E,F)** Levels of traumatin in hypocotyls **(E)** or primary leaves **(F)** of control and GmPH lines after mock- or *P. sojae* inoculation. All measurements represent the average of three independent biological replicates, 12 plants pooled per replicate. Letters represent significance groups at *p* < 0.05.

Analysis of mock-inoculated hypocotyl tissue showed that transgenic GmPH soybeans had a significantly higher level of JA-isoleucine, the bioactive form of jasmonic acid (33.2% increase). However, with *P. sojae* infection, the elevation was only 26.3% and not statistically significant (Figure 7A). Levels of jasmonic acid were elevated slightly in GmPH hypocotyl tissue for both treatments, but this was not significant (13.3% difference in mock, 11.1% difference in *P. sojae*-treated tissue, Figure 7C). Altered levels of JA-isoleucine were also seen in the uninfected primary leaf tissue distal to the site of inoculation on the hypocotyl. Levels of JA-isoleucine in control leaves showed a 44.2% decrease in *P. sojae*-inoculated tissue compared to mock (Figure 7B). This pattern was not seen in GmPH leaf tissue, where there was no change JA-Ile levels between mock- and *P. sojae*-inoculated tissue (Figure 7B). Likewise, there were no significant differences in JA levels among the leaf samples (Figure 7D). In addition to JA-Ile, there were also altered patterns of 12-hydroxyjasmonic acid and its isoleucine conjugate between control and GmPH leaf tissue (Supplementary Table 5).

The wound-induced hormone, traumatin [(10E)-12-Oxododec-10-enoic acid] (English et al., 1939; Zimmerman and Coudron, 1979; Nakashima et al., 2013), showed significantly altered patterns in both hypocotyl and leaf tissue that suggested an increased induction following *P. sojae* inoculation of GmPH plants. The increase in hypocotyl tissue was slight (13.6% between mock- and *P. sojae*-inoculated control tissue; 21.2% between mock- and *P. sojae*-inoculated GmPH tissue, Figure 7E). However in leaf tissue, the difference in levels of traumatin in both mock- and *P. sojae*-inoculated control tissues was negligible, but there was a 43.8% increase in the *P. sojae*-inoculated tissue compared to mock-inoculated GmPH tissue (*p* < 0,05; Figure 7F).

## Discussion

The objective of this study was to test whether secretion of phosphoinositide-3-phosphate (PI3P)-binding proteins could confer disease resistance on stable transgenic soybean plants. The motivation for this strategy was evidence suggesting that binding to PI3P was involved in entry of oomycete RXLR effectors and some fungal effectors into host cells (Kale et al., 2010). Although the involvement of PI3P in effector entry remains controversial (Kale et al., 2010; Petre and Kamoun, 2014; Wawra et al., 2017), Helliwell et al. (2016) showed that secretion of phosphoinositide-3-phosphate (PI3P)-binding proteins could confer resistance against oomycete and fungal pathogens on stable transgenic cacao (*T. cacao*) plants. In that study, the transgenic PI3P-binding cacao leaves were shown to have enhanced resistance to two different species of oomycete pathogens: *Phytophthora tropicalis* and *P. palmivora*, which are both causal agents of Black pod rot, along with two isolates of fungal pathogen *Colletotrichum theobromicola*, which causes a leaf and pod spot disease. Resistance could be observed when any of four different phosphoinositide-3-phosphate (PI3P)-binding proteins were expressed in the cacao leaves. Furthermore, resistance required a functional PI3P-binding site and also required secretion of the proteins to the apoplast. Those results demonstrated that the strategy was effective against diverse pathogens (Helliwell et al., 2016).

In this study, we selected two PI3P-binding proteins that would be considered acceptable in a transgenic food plant, namely the soybean PH domain protein GmPH1 and the yeast PX domain protein VAM7. As controls, we selected a VAM7 mutant that had been validated as a negative control by Helliwell et al. (2016) as well as a PI4P-binding protein FAPP1, which had also been shown to be ineffective in conferring resistance in cacao. Our results confirmed that stable expression of the two PI3P-binding proteins in soybean could increase resistance against *P. sojae*. Plants expressing GmPH protein showed resistance in both detached leaf and hypocotyl inoculation assays, while plants expressing VAM7 protein showed statistically significant resistance only in the leaf assays. Plants expressing the two proteins displayed a wide range of transcript levels for the relevant transgenes, but the level of resistance was not correlated with transcript levels. In cacao, the VAM7 constructs conferred a slightly higher level of resistance than the GmPH constructs (Helliwell et al., 2016). Differences in the level of resistance conferred might result from the level and stability of the secreted proteins in the apoplast of each species.

Here, we also examined the effect of transgene expression on nodulation by the mutualist, *B. japonicum*, which is important to soybean production and hence to the phenotype conferred by the transgenes. The results showed that two of the GmPH lines, GmPH9#4 and GmPH22#5, exhibited fewer and smaller nodules, as well as reduced physiological benefits from nodulation. Since *B. japonicum*, like other prokaryotes, does not produce PI3P, these results suggested that the physiology of the plants themselves might be responsible for the resistance to both *P. sojae* and *B. japonicum*.

To assess the physiology of the plants, we conducted transcriptome and hormone analyses. In particular, we aimed to investigate the physiological basis for the enhanced resistance against *P. sojae* and also to investigate why two of the GmPH lines, but not the VAM lines, showed increased resistance to *B. japonicum.*

Although 96 genes exhibited altered transcript levels in either the GmPH or VAM lines in the absence of *P. sojae*, only 24 exhibited altered transcript levels in both the GmPH and VAM lines. Changes shared by the two sets of lines might be associated with the PI3P-binding activities of the GmPH and VAM domain proteins produced by the lines. The GO terms “GO:0098542 Defense response to other organism” and “GO:0009753 Response to jasmonic acid” were elevated among the larger set of 96 genes, but not among the shared set of 24 genes. On the other hand, genes that responded to *P. sojae* infection in the control lines were significantly enriched in the shared set of 24 genes as well as the larger set of 96 genes. Furthermore, there was a significant correlation between the magnitude of the change during infection of the control lines and the magnitude of the change in the GmPH and VAM lines in the absence of infection. These observations suggest that expression of either of the PI3P-binding domains, but not the control constructs, may produce a physiological state related to a defense response, which perhaps better prepares the plants to resist infection. Decreased expression of an ortholog of *Arabidopsis* EDR2 in both sets of lines is consistent with this hypothesis. *Arabidopsis edr2* mutants exhibit enhanced resistance against biotrophic fungi (Tang et al., 2005; Vorwerk et al., 2007). Defense-related genes are elevated more strongly in *edr2* mutants following infection compared to wild-type plants (Tang et al., 2005; Vorwerk et al., 2007).

In the presence of *P. sojae*, a wider set of genes (56) showed greater than a 2-fold difference in both the GmPH and VAM lines than in the controls. These genes included the GO annotations “GO:0009753 Response to jasmonic acid” (12.5-fold, FDR *p* < 0.01) and “GO:0009737 Response to abscisic acid.” These observations are consistent with a stronger defense response in the GmPH and VAM lines, possibly involving altered jasmonate and abscisic acid signaling. Consistent with this observation, jasmonyl-isoleucine levels were 49.9% higher (*p* < 0.05) in mock-inoculated GmPH hypocotyls compared to the control lines. Jasmonate is well documented as being required for *Phytophthora* resistance in several plant species (Li et al., 2020; Long et al., 2021). Abscisic acid has been documented as a negative regular of plant defense against hemi-biotrophic pathogen, but can contribute positively to defense through the regulation of callose deposition (Mauch-Mani and Mauch, 2005) and also positively regulates defense against necrotrophs (García-Andrade et al., 2020). Leaf tissue distal to the site of infection exhibited elevated levels of the wounding signal traumatin and decreased levels of IAA, which can inhibit defense responses (Figure 7). Therefore, these signal compounds could also possibly contribute to elevated resistance. On the other hand, the transcriptome data did not reveal enrichment of genes annotated with “GO:0009611 Response to wounding” or “GO:0009733 Response to auxin” in any lines in the presence or absence of *P. sojae*. Fatty acid oxylipins and epoxides with anti-microbial activity (Prost et al., 2005) that were elevated in the distal leaf tissues (Supplementary Table 5) may also contribute to increased resistance.

Genes that differ in expression between the GmPH and VAM lines might account for the different levels of *P. sojae* resistance conferred by the transgenes and also the negative effects on nodulation observed in some GmPH lines but not in the VAM lines. A substantial number of genes differed more than 2-fold in transcript levels between the GmPH and VAM lines, 52 in the presence of *P. sojae* and 49 in its absence. The GO term, “GO:0098542 Defense response to other organism” was significantly enriched among both gene sets. Furthermore, genes altered during *P. sojae* infection of the control lines were significantly enriched among the 49 genes differentially altered by transgene expression in the absence of *P. sojae*. One example was an ortholog of the Arabidopsis *FMO1* gene involved in biosynthesis of L-pipecolic acid, a long-distance signal of systemic acquired resistance (elevated 161-fold by infection in the GmPH lines, but only 73-fold in the VAM lines). Although the GO term “GO:0009753 Response to jasmonic acid” was not enriched within either gene set, a key jasmonate metabolic gene, JAR1, encoding jasmonyl-isoleucine synthetase, was 5.3-fold elevated in GmPH hypocotyls in the absence of *P. sojae* compared to control lines but 3.2-fold decreased in VAM hypocotyls. Consistent with this observation, jasmonyl-isoleucine levels were 49.9% higher (*p* < 0.05) in mock-inoculated GmPH hypocotyls compared to the control lines. These observations suggest that the different levels of resistance of the GmPH lines compared to the VAM lines may derive from elevated levels of signaling compounds such as L-pipecolic acid and jasmonyl-isoleucine.

Negative effects on nodulation were observed in the two lines with the highest expression levels of the GmPH domain protein (GmPh9#4 and GmPh22#5) but not in the line (GmPH23#11) with the lowest *GmPH* gene expression, nor in the lines expressing VAM domain proteins. Jasmonate has been implicated as negative regulator of nodulation in *Lotus japonicus* and *Medicago truncatula* (Nakagawa and Kawaguchi, 2006; Sun et al., 2006), so it is possible that elevated synthesis of jasmonyl-isoleucine observed in the two lines expressing the highest levels of the GmPH domain proteins may be responsible for this negative effect. However, the fact that the VAM lines and the lowest-expressing GmPH line all exhibited elevated *P. sojae* resistance in the absence of negative effects on nodulation indicates that the two phenotypes are not inevitably connected.

Overall, the results from this study confirm the observation by Helliwell et al. (2016) that transgenic expression of PI3P-binding proteins is an effective strategy for increasing *Phytophthora* resistance in crop plants. In particular, our results show that the soybean GmPH and yeast VAM domain proteins, both from species regularly consumed by humans, are effective in this role. Our results also show that potential negative effects on interactions with beneficial microbes such as rhizobia are not an inevitable consequence of increased oomycete resistance and can be avoided. Ultimately though, additional factors may play a role in resistance when the plants are grown outside of a controlled environment. As a case in point, two small field tests conducted to date did not reveal statistically significant differences between lines expressing PI3P-binding proteins and non-transgenic soybeans for seedling establishment or yield in the presence of *P. sojae*.

The mechanisms by which secretion of PI3P-binding proteins increases resistance to *Phytophthora* infection remain to be fully resolved. Since multiple PI3P-binding proteins, but not mutant proteins, confer resistance in both soybean (this study) and cacao (Helliwell et al., 2016), the PI3P-binding activities of the proteins appear essential for producing resistance. The original rationale for employing these proteins, namely interfering with PI3P-mediated effector entry, was not directly addressed by this study, though the increased expression of many host defense genes during *P. sojae* infection could plausibly result from decreased entry of defense-suppressing effector proteins. This study does, however, demonstrate that the expression of the PI3P-binding proteins induces elevated expression of many infection-associated genes, consistent with the triggering of some kind of primed state in the plants (Mauch-Mani et al., 2017) possibly involving jasmonate (Arévalo-Marín et al., 2021). The mechanisms by which this state is produced remain to be investigated, in particular whether the PI3P molecules involved are located externally or internally. Helliwell et al. (2016) showed that PI3P-binding protein must be secreted in order to confer resistance to *Phytophthora* species. However, our experiments did not test whether PI3P-binding proteins without a secretory leader could trigger transcriptional changes associated with a possible primed state. Lu et al. (2013) also showed that secreted PI3P-binding proteins could target the mycelial surface of *P. sojae*, and Zhou et al. (2021) showed that this action could be used to target anti-microbial peptides to the surface of several *Phytophthora* species. So it is possible that secreted PI3P-binding proteins could negatively impact the pathogen directly, as a third mechanism of action.

## Data Availability Statement

The datasets presented in this study can be found in online repositories. The names of the repository/repositories and accession number(s) can be found at: NCBI GEO—GSE201739.

## Author Contributions

BT and WP conceived the project. EH, PL, JV-A, SP, EB, MK, WP, and BT planned the experiments. EH, PL, FA, MD, AC, JV-A, BK, and EB conducted the experiments. EH, BK, EB, and BT analyzed the data. EH and BT wrote the paper with input from all authors. All authors contributed to the article and approved the submitted version.

## Funding

This work was supported by NSF grant IOS-0965353 (to BT), USDA NIFA AFRI grant 2011-68004-30104 (to BT and WP), and by Oregon State University. The metabolite analyses were in part supported by USDA NIFA grant 2017-67013-26524 to MK, and the *B. japonicum* assays were in part supported by NSF-DEB-193239 to SP.

## Conflict of Interest

The authors declare that the research was conducted in the absence of any commercial or financial relationships that could be construed as a potential conflict of interest.

## Publisher’s Note

All claims expressed in this article are solely those of the authors and do not necessarily represent those of their affiliated organizations, or those of the publisher, the editors and the reviewers. Any product that may be evaluated in this article, or claim that may be made by its manufacturer, is not guaranteed or endorsed by the publisher.

## Footnotes

^1^ https://www.arabidopsis.org/

## Supplementary Material

Supplementary Figure 1 Quantitative real-time PCR (qRT-PCR) measurements of the quantity of *P. sojae* genomic DNA relative to soybean genomic DNA (*PsActin* vs. *GmCYP2*) in non-transformed cv. Williams within 48 h after inoculation. Bars represent the average of three biological replicates (six pooled plants per replicate). Errors represent the SEM.

Supplementary Figure 2 Alignment summary of RNA sequencing output to *Glycine max* genome. Left refers to the average percentage of left reads across all RNA samples that aligned to the *G. max* genome, right refers to the average percentage of right reads across all samples that aligned to the *G. max* genome, and concordant refers to the average percentage of paired reads across all genomes that aligned with the respective mate orientation to the *G. max* genome.

Supplementary Figure 3 Quantitative reverse transcriptase PCR verification of transcript levels measured by RNA sequencing analysis from mock-inoculated soybean. Transcripts measured were Glyma04g02230 (Ornithine decarboxylase-like), Glyma10g26320 (Asparagine synthase-like), Glyma18g07396 (Glutamate synthase), and Glyma02g14630 (Phosphoenolpyruvate carboxykinase). Transcripts were normalized to the internal control gene *GmCYP2* then levels in GmPH lines (GmPh9#4 and GmPh23#11) or VAM lines (VAM13 and VAM19) were compared to the Control lines (null and Vmut). RNAseq differences were derived by linear mixed model analysis. Each qRT-PCR sample assay was run in duplicate. **(A)** GmPH lines and **(B)** VAM lines.

Supplementary Table 1 Design of RNA sequencing experiment, showing the genotype, category, treatment, and number of replicates for each RNA library sequenced.

Supplemental Table 2 FASTQ run reports and summary of RNA sequencing.

Supplemental Table 3 Genes with transcript levels differing among mock-inoculated genotypes.

Supplemental Table 5 Hormone measurements in soybean lines. Letter groups denote a significant difference between Control and GmPH soybean.

Supplemental Table 6 List of primers and sequences used in this study.
