# Supplementary figures and images for: Transgenic Soybeans Expressing Phosphatidylinositol-3-Phosphate-Binding Proteins Show Enhanced Resistance Against the Oomycete Pathogen Phytophthora sojae
Source: Front Microbiol. 2022 Jun 16;13:923281. doi: 10.3389/fmicb.2022.923281 (PMC9243418; doi:10.3389/fmicb.2022.923281)

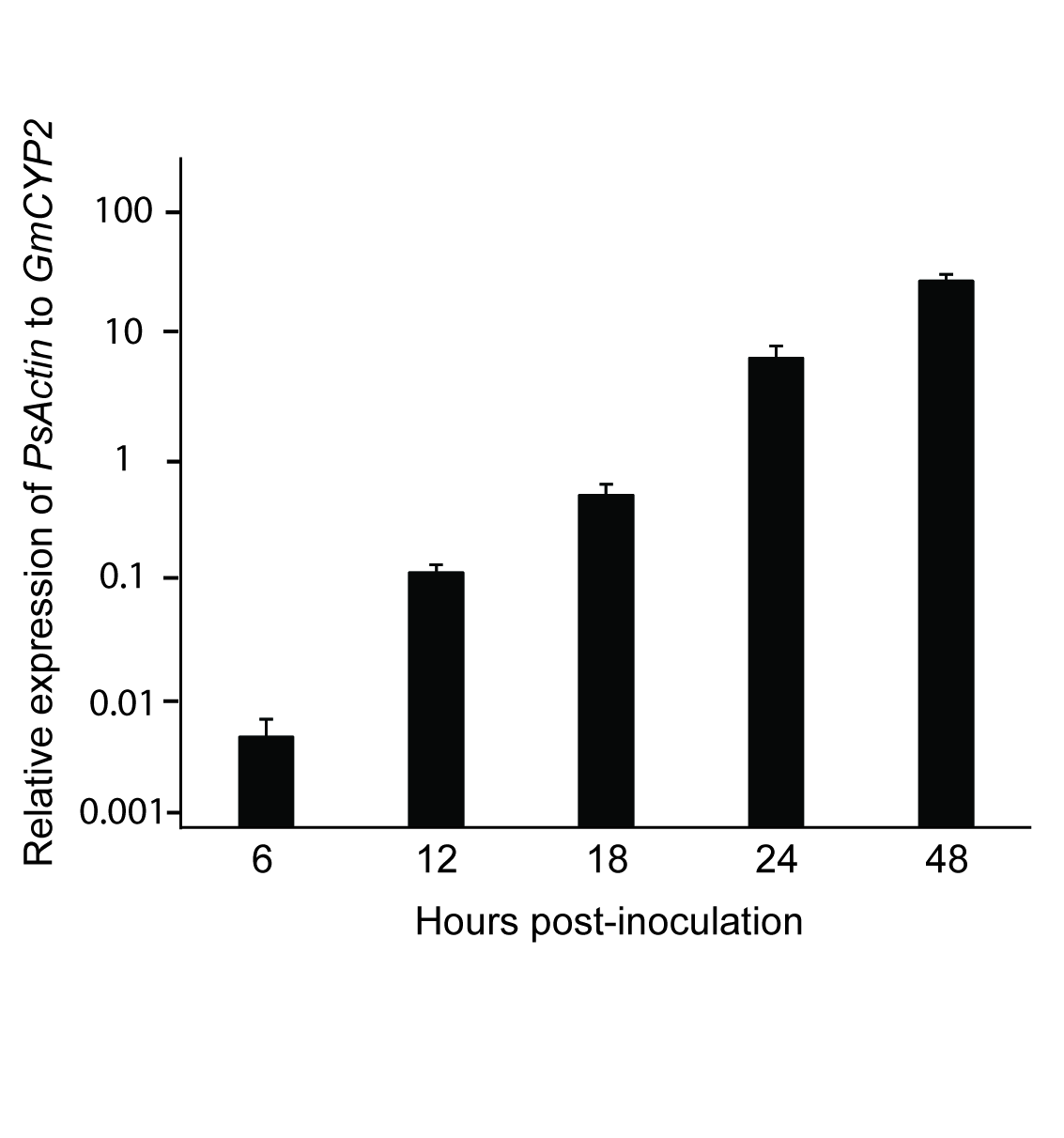

Supplement: Supplementary Figure 1 — Quantitative real-time PCR (qRT-PCR) measurements of the quantity of P. sojae genomic DNA relative to soybean genomic DNA (PsActin vs. GmCYP2) in non-transformed cv. Williams within 48 h after inoculation. Bars represent the average of three biological replicates (six pooled plants per replicate). Errors represent the SEM. [file Image_1.TIF]

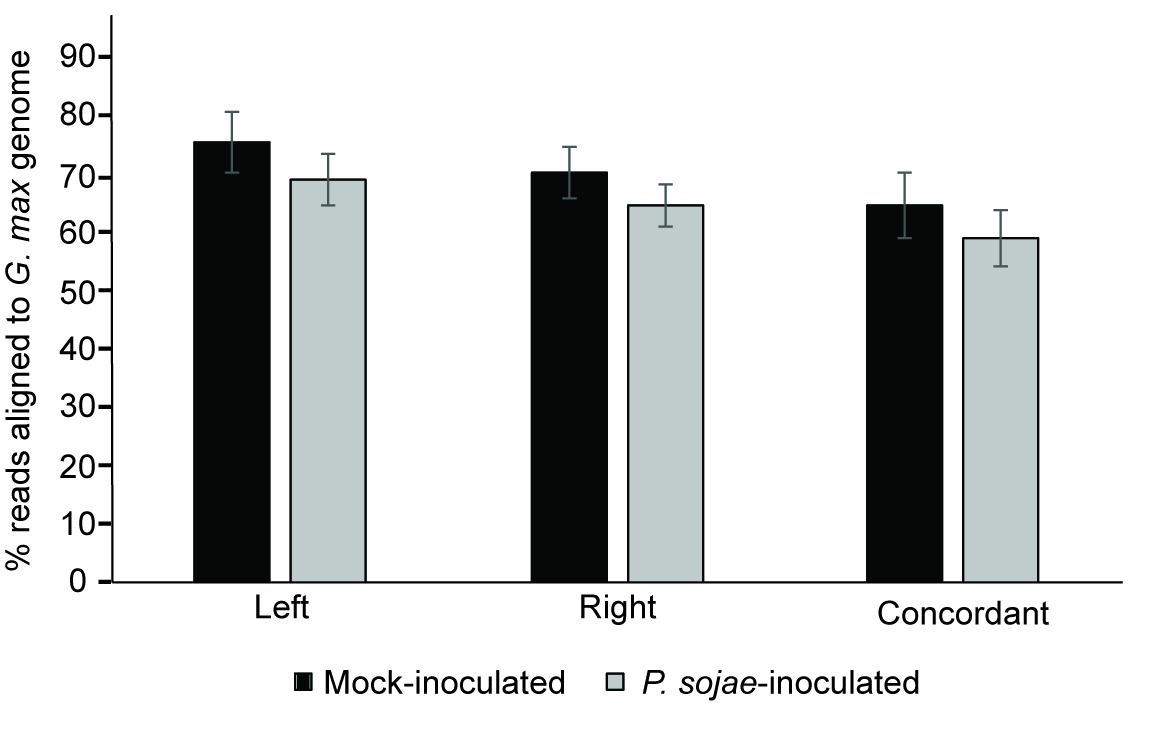

Supplement: Supplementary Figure 2 — Alignment summary of RNA sequencing output to Glycine max genome. Left refers to the average percentage of left reads across all RNA samples that aligned to the G. max genome, right refers to the average percentage of right reads across all samples that aligned to the G. max genome, and concordant refers to the average percentage of paired reads across all genomes that aligned with the respective mate orientation to the G. max genome. [file Image_2.TIF]

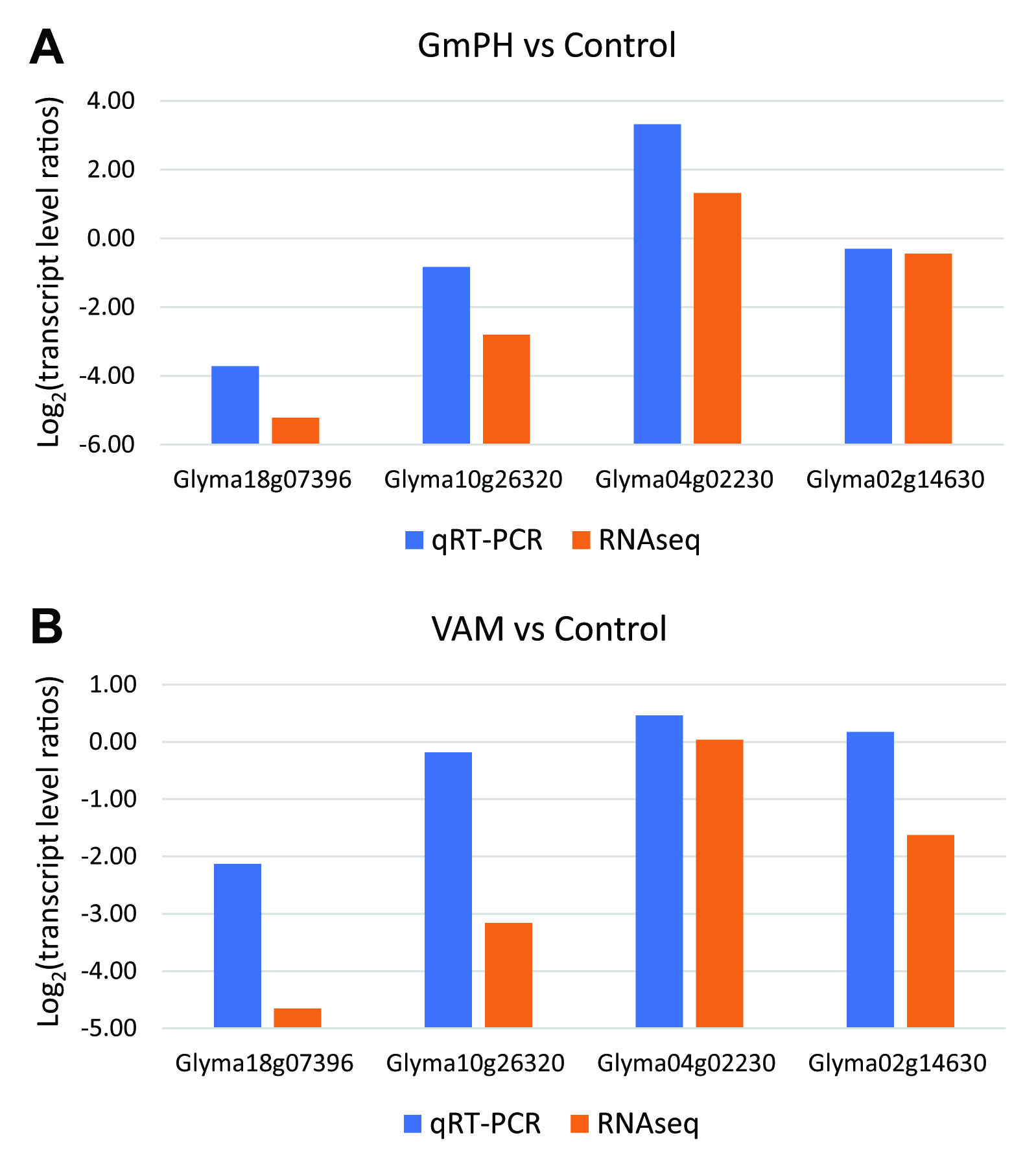

Supplement: Supplementary Figure 3 — Quantitative reverse transcriptase PCR verification of transcript levels measured by RNA sequencing analysis from mock-inoculated soybean. Transcripts measured were Glyma04g02230 (Ornithine decarboxylase-like), Glyma10g26320 (Asparagine synthase-like), Glyma18g07396 (Glutamate synthase), and Glyma02g14630 (Phosphoenolpyruvate carboxykinase). Transcripts were normalized to the internal control gene GmCYP2 then levels in GmPH lines (GmPh9#4 and GmPh23#11) or VAM lines (VAM13 and VAM19) were compared to the Control lines (null and Vmut). RNAseq differences were derived by linear mixed model analysis. Each qRT-PCR sample assay was run in duplicate. (A) GmPH lines and (B) VAM lines. [file Image_3.TIF]
